# Graft factors as determinants of postoperative delirium after liver transplantation

**DOI:** 10.1007/s13304-020-00887-3

**Published:** 2020-09-24

**Authors:** D. Patrono, F. Rigo, S. Bormida, P. Berchialla, L. Giordanengo, S. Skurzak, R. Balagna, R. Romagnoli

**Affiliations:** 1 General Surgery 2U-Liver Transplant Unit, A.O.U. Città della Salute e della Scienza di Torino, University of Turin, Corso Bramante 88-90, 10126 Turin, Italy; 2 Department of Clinical and Biological Sciences, University of Turin, Turin, Italy; 3 Regional Transplant Center, Unit of Medical Psychology for Transplantation, A.O.U. Città della Salute e della Scienza di Torino, Turin, Italy; 4 Anesthesia and Intensive Care Service 2, A.O.U. Città della Salute e della Scienza di Torino, Turin, Italy

**Keywords:** Cognitive impairment, Postoperative delirium, Surgical quality, Ageing, Outcomes, Liver transplantation

## Abstract

Post-operative delirium (POD) is a frequent complication after surgery, occurring in 15–20% of patients. POD is associated with a higher complications rate and mortality. Literature on POD after liver transplantation (LT) is limited, with the few available studies reporting an incidence of 10–47%. The aim of this study was analyzing pattern, risk factors and clinical impact of POD after LT. Data on donor and recipient characteristics, postoperative course and POD of consecutive adult LT recipients from March 2016 to May 2018 were prospectively collected and retrospectively analyzed. Risk factors for POD were analyzed using univariable logistic regression and Lasso regression. Kaplan–Meier method was used for survival analysis. 309 patients underwent LT during study period; 3 were excluded due to perioperative death. Incidence of POD was 13.4% (*n* = 41). The median day of onset was 5th (IQR [4–7]) with a median duration of 4 days (IQR [3–7]). Several risk factors, related to the severity of liver disease and graft characteristics, were identified. Graft macrovesicular steatosis was the only factor independently associated with POD at multivariable analysis (OR 1.27, CI 1.09–1.51, *p* = 0.003). POD was associated with a higher rate of severe postoperative complications and longer intensive care unit and hospital stay, but did not significantly impact on patient and graft survival. Incidence of POD after LT is comparable to that observed after general surgery and graft factors are strongly associated with its onset. These results help identifying a subset of patients to be considered for preventive interventions.

## Introduction

Post-operative delirium (POD) is one of the most common postoperative complications. Delirium can be defined as a transient, mostly reversible organic-mental syndrome that includes confusion, cognitive impairment, decline of vigilance, increased or reduced psychomotor activity and a disturbed sleep–wake cycle [1]. Prevalence in the general population is around 1–2%, whereas in hospitalized patients its incidence is 10–40%, with values reaching 56% in elderly patients and up to 87% in patients admitted to intensive care units (ICU) [1–3]. Nowadays, more and more elderly patients are considered eligible for surgery and surgical units have become familiar with the peculiarities of the ageing organism and its response to surgical stress [4, 5]. Postoperative delirium has been associated with prolonged length of ICU and hospital stay and increased costs of care, morbidity and mortality [6–8]. As incidence of POD can be reduced with specific interventions, the rate of POD has been proposed as an indirect measure of the quality of surgical care [9].

Limited data are available about POD in the setting of liver transplantation (LT). Compared to the general population of surgical patients, LT patients are younger, more frequently affected by chronic disease and are treated with immunosuppressants and steroids. Therefore, knowledge on POD gathered in the general surgical population might not be directly applicable to them. It has been suggested that pathogenesis of POD presenting after LT is multifactorial: infections, organ failure, encephalopathy and neurotoxicity of immunosuppressants have all been called into question. Reported POD incidence in LT patients ranges from 10 to 47% [10–16]. Also in the setting of LT, POD has been associated with prolonged hospital and ICU stay, and increased mortality. Previous studies have linked POD to the use of antidepressant drugs before surgery, history of pre-LT porto-systemic encephalopathy, alcohol abuse, retransplantation or high APACHE II score [10, 11, 13–16].

However, all available studies suffer from limited numerosity, lack a prospective diagnosis of POD using stringent criteria, or were performed and restricted to an ICU setting. As timely identifying patients at risk of developing POD is crucial to set up adequate precautionary interventions, the aim of this study was to analyze incidence, patterns and risk factors for POD after adult deceased-donor LT in a high-volume LT unit, using robust statistical methodology.

## Patients and methods

### Study population and design

This is a retrospective cohort study on prospectively collected data on 309 consecutive adult LT recipients transplanted at our Center in the period from March 2016 to May 2018 to identify incidence, pattern and risk factors of POD after LT. Diagnosis of POD was based on confusion assessment method (CAM) [17, 18] and was confirmed by a senior member of our dedicated team of transplant psychologists and psychiatrists who are in charge of pre-LT assessment and post-LT follow-up. In brief, patients matching POD diagnosis should present both changes of mental status characterized by acute onset and fluctuating course and inattention, and either disorganized thinking or an altered level of consciousness. For POD cases we also collected data concerning timing, duration, pharmacological treatment and associated clinical events (e.g. lines/drains/nasogastric tube self-removal, accidental falls, need for physical restraint measures, etc.). Brain imaging was not systematically obtained. For all patients, we collected data on pre-LT baseline patient characteristics (including history of alcohol abuse or hepatic encephalopathy), donor features, transplant operation (including number of packed red blood cells and other blood products units transfused, duration of surgery, end-procedure lactate) and postoperative period (including duration of mechanical ventilation, tacrolimus blood level, rejection episodes, graft function and other complications). Due to the retrospective observational nature of the study, approval by local ethics committee was not sought. All study procedures complied with the Declaration of Helsinki and the Declaration of Istanbul.

### Patient management

Indication for LT was discussed during multidisciplinary team meetings. All patients were systematically assessed by a transplant psychologist or psychiatrist before entering the waiting list. In patients with a history of alcohol abuse, a minimum of 3-months abstinence was confirmed by ethyl glucuronide hair test.

LT transplant anesthetic protocol included sedation by sodium thiopental or propofol (as induction agents), sevoflurane (for anesthesia maintenance) and analgesia using sufentanil. Patients were monitored by the mean of Entropy^®^ monitoring during surgery without specific targets unless the avoidance of values above 60. None of the patient received any premedication. Postoperative analgesia was based on continuous infusion of intra-venous morphine (usual dose between 0.6–1.2 mg/hour and stopped on postoperative day 3 or 4) and paracetamol (1000 mg/8 h).

LT was performed as a rule using piggyback technique with inferior vena cava preservation. Veno-venous by-pass was never employed. In case there was a concern for hemostasis control at the end of LT operation, patients were treated with temporary abdominal packing and delayed abdominal wall closure (“open abdomen”), as previously described [19].

Immunosuppression was based on steroids (1000 mg methylprednisolone intra-operatively; 20 mg methylprednisolone or prednisone from day 1, tapered over 3 months), tacrolimus (target level 8–10 ng/ml) and mycophenolate mofetil. Tacrolimus trough level was measured daily in inpatients and twice a week in outpatients during the first month. Induction with basiliximab (20 mg intra-operatively and on day 4) was administered in patients with autoimmune or cholestatic liver disease.

All patients were admitted to ICU after LT and discharged to the transplant ward based on clinical course and bed availability. After discharge from the hospital, they were followed-up at our outpatient clinic.

### Definitions

Early allograft dysfunction was defined according to the criteria proposed by Olthoff et al. [20]. Postreperfusion syndrome (PRS) was defined as a drop in mean arterial pressure ≥ 30% from baseline for at least one minute within 5 min from graft reperfusion [21]. Severity of PRS was graded according to Hilmi et al. as mild or severe, this last being characterized by severe hemodynamic instability with hypotension, asystole, hemodynamically significant arrhythmias, or prolonged and/or recurrent fibrinolysis [22]. Acute kidney injury (AKI) was defined and graded according to 2012 KDIGO guidelines [23]. Postoperative complications were graded according to Clavien-Dindo classification [24]. Prognostic scores, including model for-end stage liver disease (MELD), donor age * MELD (D-MELD), and donor risk-index (DRI), were calculated using formulae from the original papers [25, 26].

### Statistical analysis

Continuous variables are reported as median and interquartile range (IQR). Categorical variables are reported as number and percentage. Pre-transplant patient characteristics, donor and transplant features, as well as outcomes were compared between patients who developed POD and controls. A stratified descriptive analysis was initially performed using POD as a grouping variable. The Mann–Whitney, Chi-square test and Fisher’s exact tests were used to compare continuous and categorical variables, as appropriate. To identify variables associated with POD, univariable and multivariable logistic regression using least absolute shrinkage and selection operator (Lasso) method [27] were used. Survival analysis was performed using Kaplan–Meier method. For all analyses, the level of significance was set at 0.05. All statistical analyses were performed with R version 3.6.1. (R Foundation for Statistical Computing, Vienna, Austria. URL: https://www.R-project.org/).

## Results

**Fig. 1:**
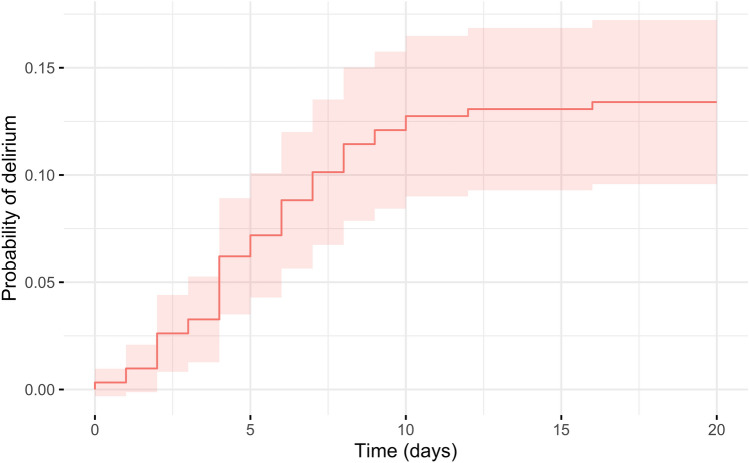
Cumulative incidence plot of delirium after liver transplantation. Light red area represents 95% confidence interval

**Fig. 2:**
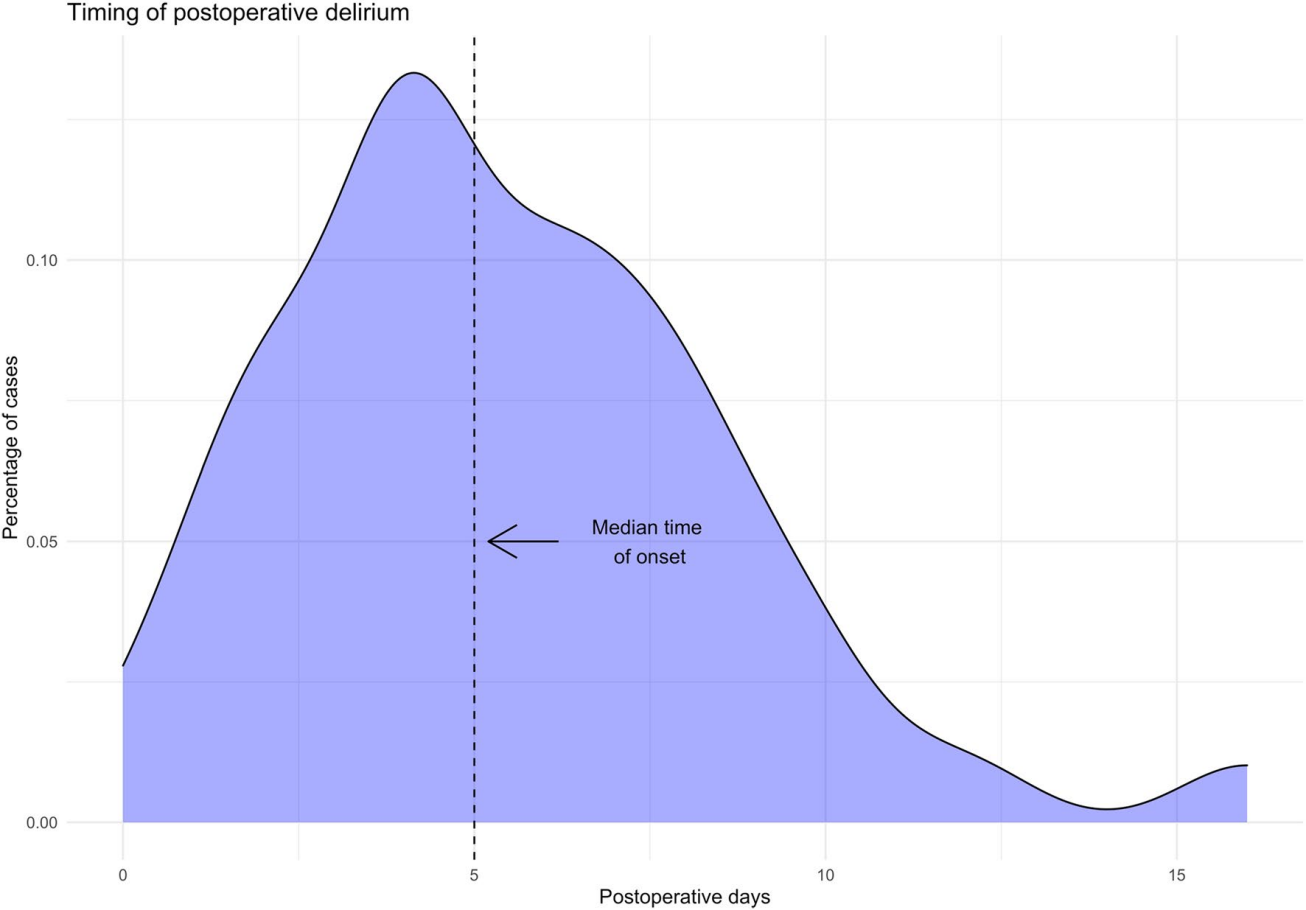
Distribution of the day of onset of postoperative delirium cases. Vertical dotted line represents median time of onset

309 LT were performed during study period, 3 of which were excluded due to on-table patient death, yielding a cohort of 306 patients for analysis. Incidence of POD was 13.4% (*n* = 41) (Fig. 1). Median timing of delirium onset and duration were 5th postoperative day (4–7) and 4 days (3–7), respectively (Fig. 2). POD was associated with an accidental fall in 3 (7.3%) patients, with nasogastric tube self-removal in 2 (4.9%) patients, with venous lines self-removal in 4 (9.8%) patients and with the need for physical restraint measures in 8 (19.5%) patients. Haloperidol and promazine were the most frequently administered treatment in 32 (78%) and 15 (36.6%) of cases, respectively. Five patients were administered a combination of two or more drugs (Table 1).

**Table 1:** Characteristics and treatment of postoperative delirium after liver transplantation

Incidence	41 (13.4%)
Timing (postoperative day)	5th (4th–7th)
Duration (days)	4 (3–7)
Associated clinical events	
Need for physical restraint measures	8 (19.5%)
Lines self-removal	4 (9.8%)
Accidental fall	3 (7.3%)
Nasogastric tube self-removal	2 (4.9%)
Treatment*	
Haloperidol	32 (78%)
Promazine	15 (36.6%)
Olanzapine	4 (9.7%)
Alprazolam	4 (9.7%)
Quetiapine	3 (7.3%)
Bromazepam	3 (7.3%)
Tiapride	2 (4.9%)
Lorazepam	2 (4.9%)
Amisulpride	1 (2.4%)
Aripiprazole	1 (2.4%)
Fluoxetine	1 (2.4%)
Lormetazepam	1 (2.4%)
Paroxetine	1 (2.4%)
Trazodone	1 (2.4%)
Zolpidem	1 (2.4%)

*5 patients were treated with a combination of two or more drugs

We observed several differences concerning baseline patient characteristics according to the development of POD (Table 2). In particular, patients who developed POD had lower pre-LT serum albumin (3.3 vs. 3.4 gr/dL) and sodium (137 vs. 140 mmol/L) levels, and higher bilirubin level (2.2 vs. 1.6 mg/dL). They were more frequently admitted in hospital (9.8% vs. 3.8%) or ICU (9.8% vs. 3.8%) before LT, were more frequently on life support (9.8% vs. 1.9%) and had more frequently a history of hepatic encephalopathy (36.6% vs .18.1%) or were encephalopathic at LT (9.8% vs. 2.6%).

**Table 2:** Patients, donor and transplant features by postoperative delirium

	No delirium (*n* = 265)	Delirium (*n* = 41)	*p*
Patients features			
Sex (male)	199 (75.1)	29 (70.7)	0.69
Recipient age (years)	55 (51–60)	57 (53–59)	0.45
Rank of LT			0.15
First	251 (94.7)	36 (87.8)	
Second	14 (5.3)	5 (12.2)	
Weight (kg)	73 (64–80)	69 (61–80)	0.35
Height (cm)	170 (165–175)	170 (164–175)	0.90
Albumin (gr/dL)	3.40 (3.0–3.9)	3.30 (2.6–3.6)	0.02
Sodium (mmol/L)	140 (136–142)	137 (134–141)	0.01
Creatinine (mg/dL)	0.84 (0.72–1.04)	0.89 (0.79–1.12)	0.21
Bilirubin (mg/dL)	1.6 (0.8–3.1)	2.2 (1.3–4.0)	0.02
INR	1.30 (1.15–1.60)	1.32 (1.21–1.58)	0.34
MELD	12 (9–7)	14 (11–17)	0.27
Previous major abdominal surgery	96 (36.2)	13 (31.7)	0.70
Renal replacement therapy before LT	12 (4.5)	3 (7.3)	0.70
Status			0.047
Home	245 (92.5)	33 (80.5)	
Hospital	10 (3.8)	4 (9.8)	
ICU	10 (3.8)	4 (9.8)	
Life support	5 (1.9)	4 (9.8)	0.02
History of encephalopathy before LT	48 (18.1)	15 (36.6)	0.01
Encephalopathy at LT	7 (2.6)	4 (9.8)	0.02
Ascites at LT	93 (35.1)	19 (46.3)	0.22
History of alcohol abuse	87 (32.8)	17 (41.5)	0.36
History of chronic HCV infection	99 (37.4)	13 (31.7)	0.60
Donor and transplant features			
Donor age (years)	63 (49–74)	65 (50–75)	0.30
Graft weight (gr)	1490 (1290–1690)	1450 (1240–1840]	0.97
Donor weight (kg)	70 (61–83)	73 (65–85)	0.53
Macrovesicular steatosis (%)	0 (0–5)	3 (0–15)	< 0.001
Microvesicular steatosis (%)	5 (0–22.5)	10 (0–20)	0.33
DRI	1.53 (1.34–2.25)	1.95 (1.38–2.38)	0.31
Total ischemia time (minutes)	422 (367–472)	445 (398–499)	0.13
Packed red blood cells transfused (ml)	1000 (500–2250)	1500 (500–2750)	0.23
Postreperfusion syndrome	34 (12.8)	10 (24.4)	0.06
End-transplant lactate (mmol/L)	2.00 (1.50–2.90)	2.40 (1.90–3.70)	0.04
Open abdomen	18 (6.8)	8 (19.5)	0.02

Data are expressed as number (%) or median (interquartile range). *LT* liver transplantation, *INR* international normalized ratio, *ITU* intensive care unit, *HCV* hepatitis C virus, *DRI* donor risk index

Patients in the POD group received a graft with a significant higher percentage of macrovesicular steatosis (3% vs. 0%), had higher levels of lactate at the end of LT (2.4 vs. 2.0 mmol/L), a higher incidence of postreperfusion syndrome (24.4% vs. 12.8%) and required more frequently temporary packing followed by a delayed second-look operation (19.5% vs. 6.8%). D-MELD was higher in POD patients (968 vs. 731). For analysis purpose, D-MELD was dichotomized into a binary variable with cutoff = 1000, which was the value associated with a steep raise in the risk of POD.

**Fig. 3:**
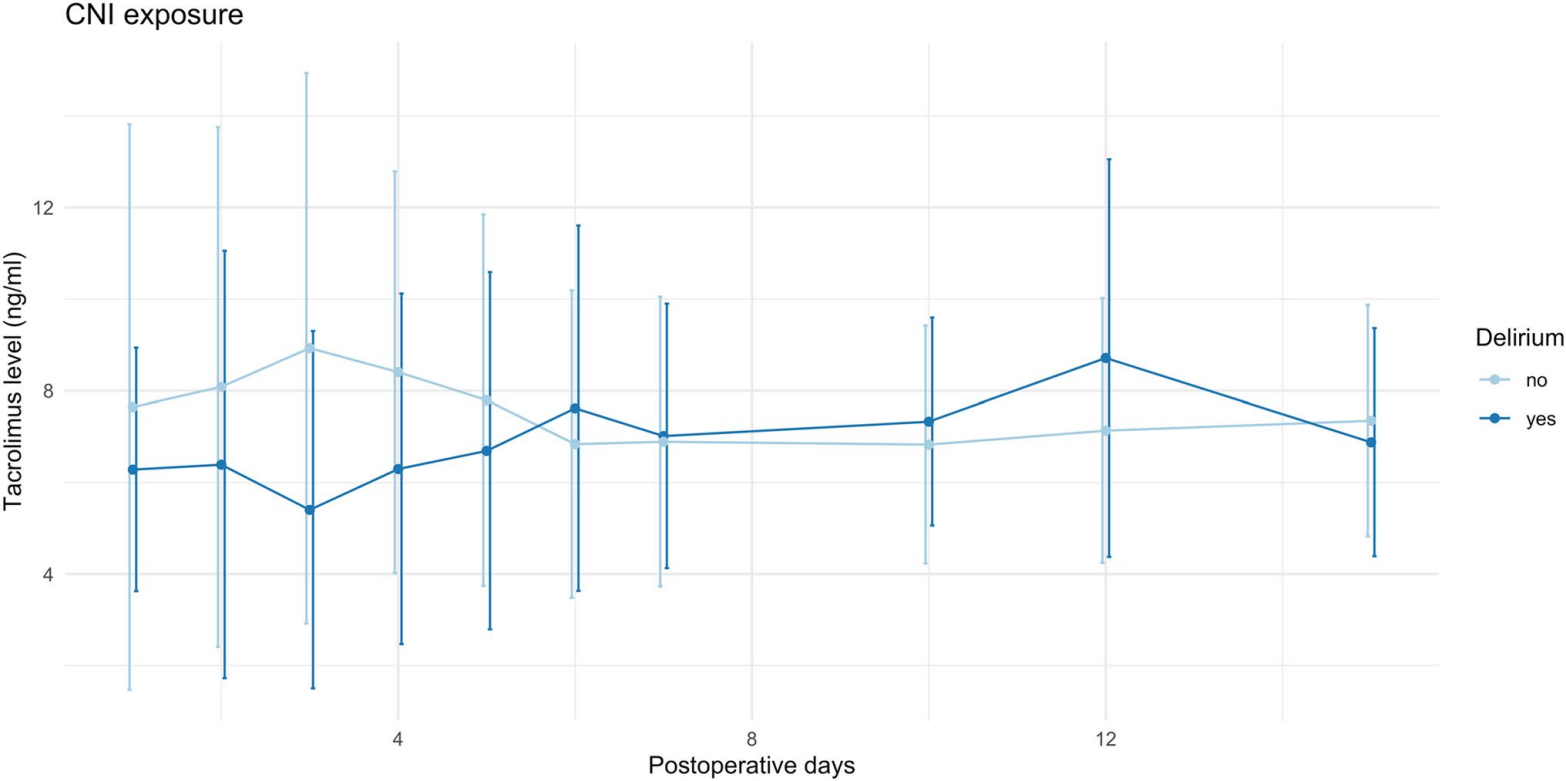
Tacrolimus levels during first 15 postoperative days according to the presence of delirium. Dots represent mean values whereas vertical error bars represent ± stadard deviation

Postoperative outcomes (Table 3) were generally inferior in patients who developed POD, as they required mechanical ventilation for longer (26 vs. 15 h) and had a higher rate of reoperation (22% vs. 10.2%) and grade III-IV surgical complications (39% vs. 16%). Both hospital (18 vs. 11 days) and ICU stay (4 vs. 3 days) were prolonged in POD patients. Noteworthy, tacrolimus through levels were not significantly different during the first 15 postoperative days (Fig. 3).

**Table 3:** Prognostic scores and postoperative variables by postoperative delirium

	No delirium (*n* = 265)	Delirium (*n* = 41)	*p*
D-MELD	731 (534–1026)	968 (649–1251)	0.02
D-MELD > 1000	0.26 (0.44)	0.46 (0.50)	0.006
Mean Tac level day 1–7 (ng/ml)	7.5 (5.1–9.6)	6.0 (4.9–8.1)	0.053
Mean Tac level day 1–15 (ng/ml)	7.5 (5.5–9.1)	6.7 (5.6–7.7)	0.25
Duration of mechanical ventilation (hours)	15 (12–26)	26 (12–55)	0.01
Early allograft dysfunction	81 (30.6)	18 (43.9)	0.13
Biopsy proven rejection	27 (10.2)	2 (4.9)	0.43
Reoperation	27 (10.2)	9 (22.0)	0.05
Complications*			0.004
No or mild complications (grade I–II)	214 (81.0)	24 (58.5)	
Severe complications (grade III–IV)	43 (16.0)	16 (39.0)	
Postoperative death (grade V)	8 (3.0)	1 (2.4)	
Grade 2–3 acute kidney injury	84 (31.7)	17 (41.5)	0.12
Renal replacement therapy	8 (3.0)	3 (7.3)	0.28
Hospital length of stay (days)	11 (9–16)	18 (14–25)	0.01
ICU length of stay (days)	3 (2–59)	4 (3–10)	0.003

Data are expressed as number (percentage) of median (interquartile range). *Clavien-Dindo classification. *D-MELD* donor age * model for end-stage liver disease score, *ICU* intensive care unit

**Fig. 4:**
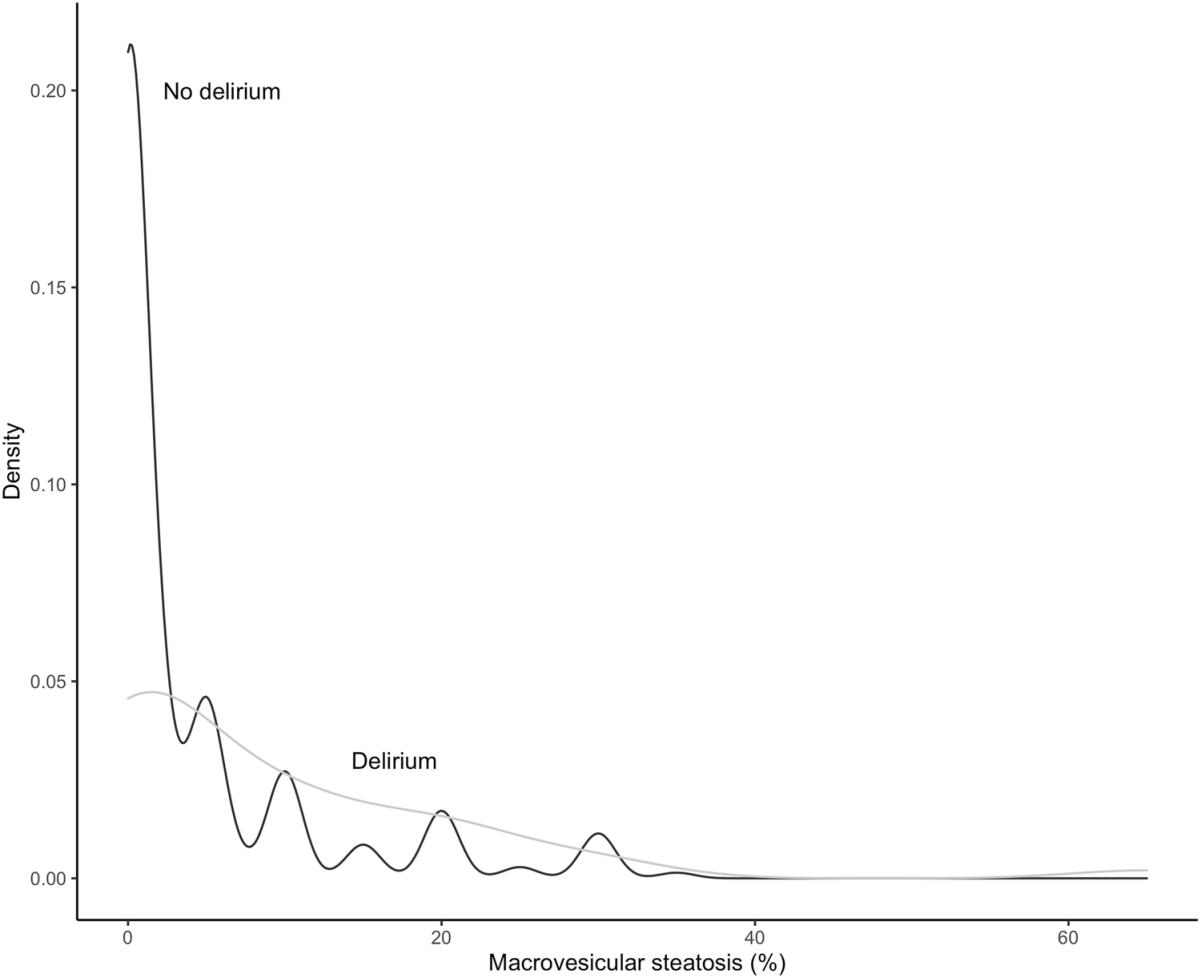
Density distribution plot of macrovesicular steatosis according to the presence of delirium

At univariable logistic regression, several variables were associated with POD (Table 4). However, of the three variables included in the multivariable Lasso model (D-MELD > 1000, history of encephalopathy and percentage of macrovesicular graft steatosis) only macrovesicular steatosis resulted significantly associated with POD (OR: 1.27, CI: 1.09–1.51, *p* = 0.003). Indeed, distribution of macrovesicular steatosis was different in patients who developed POD, confirming its association with POD (Fig. 4).

**Table 4:** Results of univariable logistic regression and multivariable Lasso regression

	Univariable logistic regression				Multivariable Lasso regression			
	Effect	Lower 0.95	Upper 0.95	*p*	Effect	Lower 0.95	Upper 0.95	*p*
Sex (female)	1.248	0.602	2.584	0.551				
Age (years)	1.144	0.817	1.602	0.433				
Retransplant	2.490	0.846	7.327	0.098				
Recipient weight (kg)	0.864	0.548	1.363	0.530				
Recipient height (cm)	1.057	0.727	1.537	0.771				
Albumin (g/dl)	0.567	0.358	0.899	0.016				
Sodium (mmol/L)	0.625	0.444	0.880	0.007				
Creatinine (mg/dl)	0.963	0.844	1.099	0.577				
Bilirubin (mg/dl)	1.096	0.999	1.203	0.053				
INR	0.955	0.751	1.214	0.707				
Previous abdominal surgery	0.817	0.404	1.652	0.574				
Dialysis	1.664	0.449	6.171	0.446				
Life support	5.622	1.444	21.884	0.013				
History of encephalopathy	2.608	1.285	5.296	0.008	1.988	0.920	4.296	0.080
Encephalopathy at LT	3.985	1.112	14.272	0.034				
Ascites	1.597	0.823	3.102	0.167				
HCV	0.778	0.385	1.573	0.485				
Alcohol	1.449	0.740	2.838	0.279				
MELD	1.224	0.851	1.762	0.276				
D-MELD	1.435	0.983	2.093	0.061				
D-MELD > 1000	2.502	1.277	4.904	0.008	1.979	0.947	4.138	0.070
DRI	1.358	0.754	2.445	0.307				
Donor weight (kg)	1.169	0.715	1.912	0.533				
Donor height (cm)	1.214	0.729	2.021	0.456				
Graft weight	1.254	0.870	1.810	0.225				
Macrovesicular steatosis (%)	1.283	1.092	1.507	0.002	1.286	1.092	1.514	0.003
Microvesicular steatosis (%)	1.051	0.742	1.489	0.779				
Cold ischemia time (min)	1.482	0.976	2.252	0.065				
PRBC (ml)	1.147	0.943	1.395	0.170				
Surgery time (min)	1.186	0.788	1.785	0.412				
End-of-transplant lactate (mmol/L)	1.258	0.945	1.676	0.116				
Mechanical ventilation time (hours)	1.127	1.022	1.242	0.016				
ICU stay (days)	1.418	1.134	1.772	0.002				
Mean Tac level day 1–7 (ng/ml)	0.537	0.283	1.021	0.058				
Mean Tac level day 1–15 (ng/ml)	0.676	0.374	1.223	0.196				

Analysis of variables associated with postoperative delirium after liver transplantation. *INR* international normalized ratio, *HCV* hepatitis C virus, *MELD* model for end-stage liver disease, *D-MELD* donor age * MELD, *DRI* donor risk index, *PRBC* packed red blood cells, *ICU* intensive care unit, *Tac* tacrolimus

**Fig. 5:**
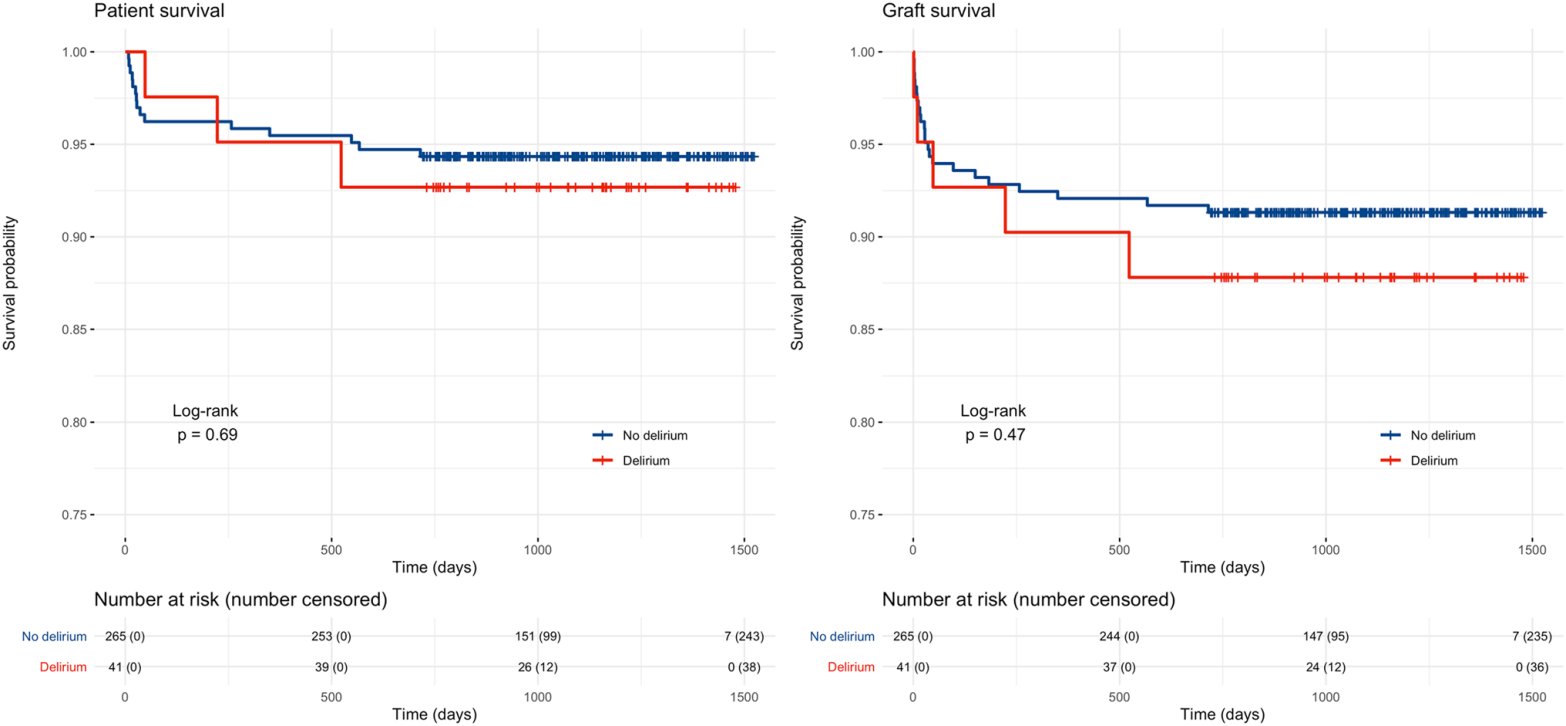
Kaplan–Meier plots for patient and graft survival

Finally, survival analysis showed a trend towards inferior survival in patients developing POD (Fig. 5). However, this finding did not reach statistical significance.

## Discussion

In this study, we linked POD delirium to several risk factors, confirming its multifactorial nature. The originality of our analysis is mainly represented by the inclusion of donor and transplant operation features in the analysis, which have been surprisingly neglected by previous literature on the subject (Table 5). To the best of our knowledge, this is the first study highlighting that donor factors, in particular the degree of graft macrovesicular steatosis, play a major role in determining the onset of POD after LT.

**Table 5:** Literature on postoperative delirium after liver transplantation

Author, year	Setting	*N*	Diagnosis	Incidence	Risk factors
Buis et al. (2002)	Deceased-donor LT	87	Not specified	25.5%	Incidence of POD was 48% in alcoholic patients versus 16% in HCV patients; shorter duration of sobriety, high pre-LT ammonia and reduced creatinine clearance associated with POD
Dhar et al. (2008)	Deceased-donor LT	101	Not specified	27.7%	Hepatic encephalopathy in the immediate preoperative period
Chiu et al. (2009)	Deceased-donor LT	30	Psychiatric consultant	27.7%* (among patients referred for psychiatric advice)	Not evaluated
Lescot et al. (2013)	Deceased-donor LT, ICU	281	POD suspicion raised by attending nurse and confirmed by senior intensivist	10%	RRT before LT; history of encephalopathy grade ≥ 2; PRBC transfusion
Wang et al. (2014)	Living-donor LT, ICU	78	CAM-ICU	47.4%	History of alcohol abuse of hepatic encephalopathy; APACHE II score ≥ 16; orotracheal intubation ≥ 5 days
Bhattacharya et al. (2017)	Deceased and living-donor LT	144	Delirium Observation Screening scale 8	25%	Age; MELD; pre-LT hospital admission; alcohol abuse; duration of mechanical ventilation; urinary tract infection; pneumonia; combined transplantation
Oliver et al. (2017)	Deceased-donor LT,	181	Retrospective search of electronic records	21%	Antidepressants and encephalopathy prior to LT

*N* number of cases, *Lt* liver transplantation, *ICU* intensive care unit, *POD* postoperative delirium, *RRT* renal replacement therapy, *PRBC* packed red blood cells, *CAM* confusion assessment method, *APACHE* acute physiology and chronic health disease classification system, *HCV* hepatitis C virus, *MELD* model for end-stage liver disease

Looking at our data, the whole picture appears rather clear. POD incidence was higher in patients with signs of more severely compromised hepatic function (lower sodium and higher bilirubin levels, history of encephalopathy), poor nutritional status (lower albumin) and who were more frequently admitted to hospital or on life support prior to LT. These patients also showed higher incidence of postreperfusion syndrome and higher lactate levels at the end of transplant operation, which are early signs of impaired graft function [28, 29], a finding which in turn is in keeping with the association of POD with higher D-MELD and graft steatosis, this last being a well-known risk factor of poor graft function after LT [30]. Finally, patients suffering from POD also presented an increased rate of reoperation, had a higher rate of severe postoperative complications and had longer ICU and hospital stay. Taken as a whole, these findings suggest that delirium represents a hallmark of a more deranged physiology before and after LT. Sicker patients receiving suboptimal grafts and suffering from postoperative complications would be at particularly high risk of developing POD. These different risk factors appear to be closely interlinked and likely act in an additive way. Surprisingly, multivariable analysis identified macrovesicular steatosis as the only variable independently associated with POD, suggesting that graft quality and function is pivotal in determining POD after LT. Although the minimal median difference in the degree of macrovesicular steatosis between study groups (0% vs. 3%) could seem clinically insignificant, it should be noted that this difference reflected a completely different distribution of this variable, as shown in Fig. 4.

In part, the concept that POD rate can be higher in sicker patients has been suggested by previous studies. Lescot et al. [14] identified renal replacement therapy, history of encephalopathy and packed red blood cells transfusion as risk factors for POD, highlighting the relevance of intraoperative events, along with patient history. In a study on the incidence of POD in the setting of ICU after living donor LT by Wang et al. [16] patients with APACHE II score ≥ 16 or who were on mechanical ventilation for ≥ 5 days were at higher risk of POD. The role of mechanical ventilation was confirmed by Bhattacharya et al. [10], who also found an association of POD with age, MELD, pre-LT hospital admission, history of alcohol abuse, urinary and pulmonary infection, and combined transplantation. Overall, these findings are in keeping with the hypothesis that POD may represent a response to surgical and physiological stress. In this view, POD could be frequently interpreted as an epiphenomenon of poor pre-LT status or a complicated postoperative course. Our analysis confirms this concept and takes it one step further by linking POD to graft factors, which are major determinants of postoperative course after LT.

Incidence of POD in our series was 13.4%, which is lower as compared to previous series in the setting of LT [10–16], but comparable to the figure in the general surgical population [9, 31, 32]. This discrepancy is likely due to the different diagnostic modalities employed in previous studies, which is also reflected by the high variability of POD incidence across different series (Table 5). In contrast with previous studies [10, 11, 16], we did not identify history of alcohol consumption before LT as a risk factor for LT. This can be explained by our rigorous selection policy for transplant candidates with alcoholic cirrhosis, who are closely followed-up during the evaluation process and while on the waiting list. Also, patients presenting with symptoms of abstinence from alcohol after LT were not classified as having POD unless they matched aforementioned diagnostic criteria for POD. Unfortunately, data about duration of alcohol abstinence before LT and concomitant use of other recreational drugs, which may have allowed fine-tuning our analysis, were not available.

As POD has been associated with reduced 1-year survival and long-term cognitive impairment [6–8], early identification of patients at increased risk for POD and implementation of mitigation or prevention strategies is of paramount importance. Environmental interventions like early discharge from ICU, resumption of oral feeding, lines and drains removal and free access to the ward for patient’s families are widely adopted in our unit. Among pharmacological interventions, sedation with dexmedetomidine, an α2-adrenoceptor agonist, has shown promising results in the general population [33]. However, data are lacking in the setting of LT and, as dexmedetomidine has prevalent hepatic metabolism [34], its use could be problematic in the early postoperative course after LT.

Limitations of our study include its retrospective, single-center nature. As aforementioned, limited information about duration of alcohol abstinence, social background and associated use of other substances might have precluded finding a significant association of POD with history of alcohol abuse.

## Conclusion

In conclusion, POD after LT is associated with severity of pre-LT hepatic disease and with graft features. In particular, graft steatosis in the only factor independently associated with POD. Incidence of POD is higher in patients suffering from severe complications and with a prolonged postoperative stay. Further studies are needed to identify strategies to prevent POD after LT.

## Data Availability

The data that support the findings of this study are available on request from the corresponding author. The data are not publicly available due to privacy restrictions.
